# Congestion of Lungs and Its Dangers

**Published:** 1890-02

**Authors:** 


CONGESTION OF THE LUNGS AND ITS DANGERS. By Thomas Nichol, M. D., LL. D., D. C. L.

This is No. 6 of "Montreal Tracts on Homœopathy." Dr. Nichol calls special attention to numbers of cases of this affection which are mistaken for heart affections. It is a valuable, practical paper, and its low price, ten cents, should constrain every physician to send to "The Montreal Homœopathic Pharmacy" for a copy.

